# Identifying risk factors for recurrence of papillary thyroid cancer in patients who underwent modified radical neck dissection

**DOI:** 10.1186/s12957-018-1496-1

**Published:** 2018-10-12

**Authors:** Young Jae Ryu, Jin Seong Cho, Jung Han Yoon, Min Ho Park

**Affiliations:** Department of Surgery, Chonnam National University Medical School, 322 Seoyang-ro Hwasun-eup, Hwasun-gun Jeonnam, Gwangju, 58128 South Korea

**Keywords:** Papillary thyroid cancer, Modified radical neck dissection, Recurrence

## Abstract

**Background:**

Papillary thyroid cancer (PTC) patients with ipsilateral neck metastatic lymph node (LN) and those with contralateral neck metastatic LN belong to N1b. Only a few studies have reported on comparisons with regard to laterality of metastatic lateral LN. The aim of this study was to evaluate predictive factors for contralateral neck LN metastasis and to determine prognostic factors for recurrence in PTC patients with N1b.

**Methods:**

This retrospective study reviewed the medical records of 390 PTC patients who underwent total thyroidectomy and central LN dissection plus ipsilateral or bilateral modified radical neck dissection (MRND) between January 2004 and December 2012.

**Results:**

During a median follow-up of 81 (range, 6–156) months, 84 patients had a recurrence in any lesion. Male gender, a main tumor of more than 2 cm, number of metastatic central LN, number of harvested and metastatic lateral LN, total LN ratio, multifocality, bilaterality, and gross ETE had significance in the patients who underwent bilateral MRND. In multivariate analysis according to recurrence, patients with LN ratio > 0.44 in the central compartment (hazard ratio [HR], 1.890; 95% confidence interval [CI], 1.124–3.178; *p* = 0.015), LN ratio > 0.29 in the lateral compartment (HR, 2.351; 95% CI, 1.477–3.743; *p* < 0.001), and multifocality (HR, 1.583; 95% CI, 1.030–2.431; *p* = 0.036) were associated with worse RFS. However, the type of MRND was statistically significant only in univariate analysis.

**Conclusions:**

Recurrence in N1b PTC patients is predicted by central neck LN ratio > 0.44, lateral neck LN ratio > 0.29, and multifocality of tumors. We suggest that patients with these factors should receive short-term follow-up using image modalities like ultrasonography and computed tomography.

## Background

Papillary thyroid carcinoma (PTC) is the most common histologic type of thyroid cancer, and its incidence has been increasing worldwide. The prognosis for PTC is better than for other types of thyroid cancer; however, the involvement of lymph nodes (LNs) is up to 80% at diagnosis [1]. It is generally accepted that the spread pattern of LN in PTC is central compartment, ipsilateral compartment, and contralateral compartment sequentially. Although the most common location of LN involvement is the central compartment, skip metastasis (lateral LN metastasis without central LN metastasis) may be observed [2]. The definition of regional LN distinguishes between N1a (levels VI, VII) and N1b (levels I, II, III, IV, V, or retropharyngeal nodes); nevertheless, recent TNM staging did not consider the location of LN involvement [3]. The size of metastatic lateral LNs in surgical specimens is often bigger than is seen with metastatic central LN; however, it is not clear whether or not the reason behind poor outcomes for PTC patients with N1b is location, size, or number of metastatic LN. Several studies revealed that patients with pathologic N1b had a worse prognosis than those with pathologic N1a [4–6]. In addition, some authors reported that the patients with N1b disease had poorer disease-specific survival than those with N0 or N1a and the cause of death is due to distant metastasis rather than locoregional metastasis [7].

There is still debate around performing prophylactic central LN dissection for clinically LN negative PTC patients; however, it is not acceptable performing prophylactic lateral LN dissection for PTC patients without clinical N1b disease. According to recent American Thyroid Association (ATA) guidelines, comprehensive modified radical neck dissection (MRND) encompassing levels II–V was recommended for patients who were clinically N1b [8]. Few studies have compared clinocopathologic characteristics between ipsilateral MRND and bilateral MRND. Ohshima et al. reported that patients who underwent thyroidectomy and bilateral MRND had better 10-year survival rate (97.1% vs. 83.7%) and lower cancer death (5.8% vs. 28.1%) than those who underwent thyroidectomy and ipsilateral MRND [9]. On the other hand, Ito et al. revealed that N1b PTC patients, regardless of type of MRND, with metastatic lateral LNs smaller than 3 cm, with less than five metastatic lateral LNs, or without extranodal extension had similar survival outcomes compared with those N1a PTC patients [10].

The potential for detecting suspicious lateral LN with ultrasonography (US) and computed tomography (CT) is higher than that of central LN [11]. However, if suspicious contralateral LN remains, then residual or persistent disease can have potential effects on postoperative management. Thus, the aim of this study was to evaluate predictive factors for contralateral LN metastasis in PTC patients who underwent total thyroidectomy and central LN dissection, plus ipsilateral or bilateral MRND. Also, we wished to determine prognostic factors for recurrence in PTC patients with N1b.

## Methods

### Patients’ population

We reviewed the medical records of 9135 patients who underwent thyroid surgery at Chonnam National University Hwasun Hospital between January 2004 and December 2012. Exclusion criteria were as follows: patients who had less than a 6-month follow-up period, who underwent reoperation due to suspicious residual tumor or LN within 6 months of initial surgery, who underwent thyroid surgery for reasons other than PTC, who had discordant histology between thyroid tumor and LN on the pathologic report, who did not undergo comprehensive LN dissection in the lateral neck compartment, who had only contralateral lateral metastatic LN, who did not undergo thyroidectomy and MRND concurrently, who did not achieve R0 resection, who had secondary malignancy during follow-up, who had distant metastasis at initial diagnosis, and who had abnormal thyroid function test before first surgery. We enrolled a total 390 patients who underwent total thyroidectomy and central LN dissection plus ipsilateral or bilateral MRND in this study. This retrospective study was approved by the institutional review board in our hospital.

### Operation

All patients were examined by neck US and neck CT during preoperative evaluation to scheme surgical extent, and especially to check the lateral neck compartment. We performed prophylactic central neck dissection, while MRND was not performed in the case of absence of evidence in the lateral compartment. When suspicious lateral LN was detected, fine needle aspiration cytology (FNAC) revealed the presence of absence of LN metastasis. However, in cases of uncertainty with FNAC, we performed an excisional frozen biopsy during the operation to proceed with MRND. Therefore, all patients in this study underwent therapeutic MRND. The performed surgeries included total thyroidectomy, central neck dissection, and MRND in a sequential manner. Central neck refers to level VI (pretracheal, paratracheal, prelaryngeal) or level VII (upper mediastinal LN). MRND refers to comprehensive excision of neck levels II–V with preservation of more than one in three structures: spinal accessory nerve, internal jugular vein, and sternocleidomastoid muscle. Level I dissection was not performed because it is a rare event and preoperative image modalities did not detect suspicious level I LN in enrolled patients. The boundary of LN levels were divided by operator and sent to the department of pathology. All patients were inserted with a drain after procedure.

### Histopathologic examination

Surgical specimens were examined by more than two experienced pathologists. The main tumor was defined as the largest tumor. The laterality of ipsilateral MRND was consistent with the location of the main tumor. LN ratio was defined as the number of metastatic LN divided by the number of harvested LN, and skip metastases was indicated lateral LN metastasis without central LN metastasis. TNM stage and ETE were reclassified according to recent American Joint Committee on Cancer (AJCC) recommendations [3].

### Postoperative follow-up

All patients received 30–100 mCi of radioactive iodine therapy 2–3 months after surgery because most patients had the possibility of more than an intermediate risk of structural disease recurrence according to recent ATA management guidelines. The patients were followed up every 3 to 6 months for 5 years and annually thereafter, if exhibiting no evidence of disease. All patients also received regular physical examination, neck US, chest radiography, whole-body iodine scanning, measurement of serum-free thyroxine, thyrotropin, thyroglobulin (Tg), and anti-thyrogobulin antibody concentrations. We defined recurrence as structural recurrence. Locoregional recurrence was confirmed by FNAC based on the result of imaging modalities such as neck US, neck CT, 18F-fluorodeoxyglucose positron emission tomography CT, and whole-body scan. Distant metastasis was confirmed by the abovementioned imaging modalities. Most patients with structural recurrence underwent reoperation; however, if the patients had an unresectable lesion or distant metastasis, radioactive iodine therapy was considered as a first option.

### Complications

Hypoparathyroidism and recurrent laryngeal nerve palsy were classified as transient or permanent based on 6 months after surgery. We defined hypoparathyroidism as postoperative serum parathyroid hormone level below normal, with a concomitant low calcium level and requiring calcium and vitamin D supplementation. Patients who underwent thyroid surgery in our institution underwent examination for the level of PTH in 6 h, 24 h, and 48 h postoperatively. Patients who had a lower level of PTH were checked every 2 days during admission. The level of PTH was examined with the level of total calcium and ionized calcium. We considered recurrent laryngeal nerve palsy through flexible laryngoscopy as well as voice change after surgery. All patients in this study were not routinely examined via preoperative laryngoscopy. However, patients who had suspicious gross ETE into posterior surface of the thyroid or trachea or who had symptoms related with voice change underwent preoperative laryngoscopy. Postoperative laryngoscopy was performed selectively for patients who had preoperative experience and with symptoms regarding voice change, or with suspicion of recurrent laryngeal nerve injury. Postoperative bleeding was defined as the case which underwent the operation, and chyle leakage was defined as the case which underwent operative or conservative management.

### Statistics

Disease-specific mortality was a rare event. Therefore, the primary end point was recurrence in any lesion. We defined recurrence-free survival (RFS) as the time between the first operation and confirmation of recurrence. Continuous variables are represented as median (range) or mean (standard deviation, SD), while categorical variables are shown as a number (percent). Independent *t* test and chi-square analysis were used to compare between ipsilateral MRND and bilateral MRND. A univariate Cox proportional hazards model was used to analyze the relationship between clinicopathologic variables and recurrence-free survival. Multivariate Cox proportional hazards regression analyses by way of backward elimination were performed using the variables with *p* values < 0.05 in the univariate analyses. The receiver operating characteristic curve was used to calculate optimal value of LN ratio in the central and the lateral compartment. We used the log-rank test and the Kaplan-Meier curve to calculate differences in RFS. We performed all statistical analyses using SPSS version 23.0 (IBM Inc., Armonk, NY, USA) and defined statistical significance as *p* less than 0.05.

## Results

### Patients’ demographics

Of a total 390 patients, median age (range) was 46 years (17–80) and 118 patients (30.3%) were male. Patients who underwent ipsilateral MRND and bilateral MRND were 346 (88.7%) and 44 (11.3%), respectively. Mean (SD) size of the main tumor was 1.61 cm (± 0.97) and patients in which the main tumor was more than 2 cm were 93 (23.8%). Findings for T stage were as follows: T1a, 121 patients (31.0%); T1b, 109 (27.9); T2, 40 (10.3%); T3a, 4 (1.0%), T3b, 52 (13.3%); and T4a, 64 (16.4%). One hundred forty-two (36.4%) and 125 (32.1%) patients had multifocality and bilaterality of tumors. Seventy-five (19.2%) patients had minor ETE, and 116 (29.7%) patients had gross ETE of the main tumor. Mean (SD) number of harvested central LN and metastatic central LN were 7.4 (± 6.0) and 3.8 (± 4.0). Mean (SD) number of harvested central LN and metastatic lateral LN were 18.6 (± 10.3) and 4.9 (± 3.9). Skip metastasis showed in 86 (22.1%) patients. Patients with stage I were 285 (73.1%); stage II, 79 (20.3%); and stage III, 26 (6.7%). We observed chronic lymphocytic thyroiditis (CLT) in 100 (25.6%) while 20 (5.1%) patients showed lymphovascular invasion (LVI). Median follow-up was 81 (range, 6–156) months (Table 1).

**Table 1:** Patients’ demographics

Variables	Number (%)
Age (years)^§^	46 (17–80)
≤ 55 years	285 (73.1)
Male	118 (30.3)
Hypertension	62 (15.9)
Diabetes	26 (6.7)
Modified radical neck dissection	
Ipsilateral	346 (88.7)
Bilateral	44 (11.3)
Main tumor size (cm)^*^	1.61 ± 0.97
> 2 cm	93 (23.8)
T stage	
T1a	121 (31.0)
T1b	109 (27.9)
T2	40 (10.3)
T3a	4 (1.0)
T3b	52 (13.3)
T4a	64 (16.4)
Multifocality	142 (36.4)
Bilaterality	125 (32.1)
Extrathyroidal extension	
No	199 (51.0)
Minor	75 (19.2)
Gross	116 (29.7)
Number of central lymph node	
Harvested^§, *^	6 (2–65), 7.4 ± 6.0
Metastatic^§, *^	3 (0–29), 3.8 ± 4.0
Number of lateral lymph node	
Harvested^§, *^	16 (8–62), 18.6 ± 10.3
Metastatic^§, *^	4 (1–23), 4.9 ± 3.9
Skip metastases	86 (22.1)
Stage	
I	285 (73.1)
II	79 (20.3)
III	26 (6.7)
Lymphovascular invasion	20 (5.1)
Chronic lymphocytic thyroiditis	100 (25.6)
Recurrence	84 (21.5)
Follow-up^§^	81 months (6–156)
Total patients	390

^§^Median and range

*Mean and standard deviation

### Recurrence

Eighty-four (21.5%) patients demonstrated recurrence during the follow-up period. Among the 33 patients with recurrence in the central compartment or operative bed, 12 patients had recurrence in the lateral compartment; 1 patient, in the distant lesion; and 1 patient, in the lateral compartment and distant lesion. Forty-eight patients had recurrence only in the lateral compartment. Three patients showed only distant metastasis. The most common organ of distant metastasis is the lung (4 patients) followed by the bone (1 patient) (Table 2). Of 84 patients who had a recurrence, 79 (94.0%) patients had a recurrence within 5 years after surgery.

**Table 2:** Distribution of recurrence site

	Number
Central LN or operative bed	33
Only central LN or op bed	19
+ Lateral LN	12
+ Distant metastasis	1
+ Lateral LN + distant metastasis	1
Only lateral LN	48
Only distant metastasis	3

*LN* lymph node

### Comparison according to MRND type

Between patients who underwent unilateral MRND and the patients with bilateral MRND, male gender, larger than a 2 cm main tumor, number of metastatic central LN, number of harvested and metastatic lateral LN, total LN ratio, multifocality, bilaterality, and gross ETE had significance in patients who underwent bilateral MRND. There was no statistical association with age, CLT, LVI, and TNM stage (Table 3).

**Table 3:** Comparison of MRND type and clinicopathologic characteristics

Variables	Ipsilateral MRND	Bilateral MRND	*p*
	*N* = 346	*N* = 44	
Age	46.2 ± 13.3	48.1 ± 15.6	0.369
≤ 55 years	255 (73.7)	30 (68.2)	0.471
> 55 years	91 (26.3)	14 (31.8)	
Sex			< 0.001
Female	252 (72.8)	20 (45.5)	
Male	94 (27.2)	24 (54.5)	
Main tumor size (cm)	1.54 ± 0.91	2.20 ± 1.21	< 0.001
≤ 2 cm	272 (78.6)	25 (56.8)	0.002
> 2 cm	74 (21.4)	19 (43.2)	
Number of central lymph node			
Harvested	7.2 ± 5.9	8.4 ± 6.2	0.210
Metastatic	3.4 ± 3.5	6.3 ± 6.0	< 0.001
Number of lateral lymph node			< 0.001
Harvested	16.6 ± 8.1	33.6 ± 13.3	< 0.001
Metastatic	4.2 ± 2.9	10.5 ± 5.5	
Skip metastases	80 (23.1)	6 (13.6)	0.179
LN ratio	0.34 ± 0.19	0.41 ± 0.21	0.012
Multifocality	114 (32.9)	28 (63.6)	< 0.001
Bilaterality	101 (29.2)	24 (54.5)	0.001
ETE			0.003
No/minor	252 (72.8)	22 (50.0)	
Gross	94 (27.2)	22 (50.0)	
CLT	92 (26.6)	8 (18.2)	0.274
LVI	16 (4.6)	4 (9.1)	0.263
Stage			0.406
I	255 (73.7)	30 (68.2)	
II	70 (20.2)	9 (20.5)	
III	21 (6.1)	5 (11.4)	
Recurrence	67 (19.4)	17 (38.6)	0.006

*MRND* modified lateral neck dissection, *LN* lymph node, *ETE* extrathyroidal extension, *CLT* chronic lymphocytic thyroiditis, *LVI* lymphovascular invasion

### Uni- and multivariate analyses according to recurrence

**Fig. 1:**
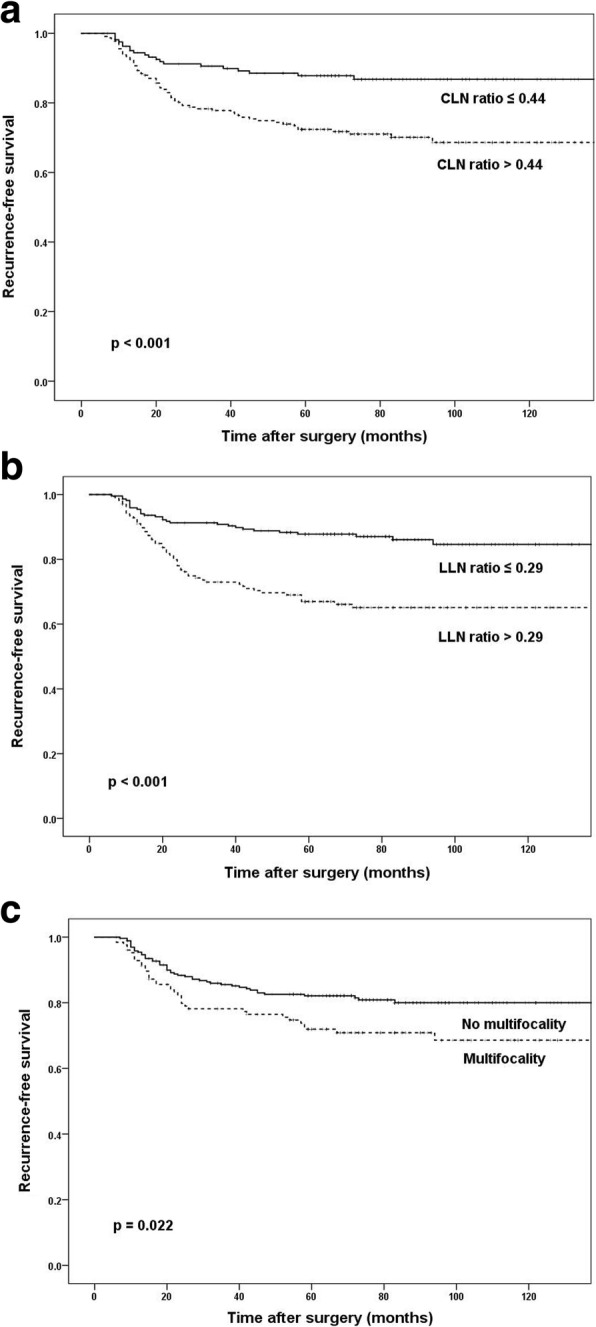
Kaplan-Meier curves according to CLN ratio (**a**), LLN ratio (**b**), and multifocality (**c**). CLN central neck lymph node, LLN lateral neck lymph node

In univariate analysis of associations with recurrence, larger than 2 cm main tumor (*p* = 0.025), LN ratio > 0.44 in the central compartment (*p* < 0.001) and LN ratio > 0.29 in the lateral compartment (*p* < 0.001), bilateral MRND (*p* = 0.004), multifocality (*p* = 0.012), no CLT (*p* = 0.037), and gross ETE (*p* = 0.040) showed statistically significant differences. However, there were no differences in RFS with age, sex, skip metastasis, bilaterality, LVI, or stage (Table 4, Fig. 1a−c).

**Table 4:** Univariate analysis of risk factors for recurrence

	Exp (B)	95% CI for Exp (B)	*p*
Age			
≤ 55 years	1		
> 55 years	1.138	0.713–1.816	0.589
Sex			
Female	1		
Male	1.037	0.653–1.648	0.877
Main tumor size			
≤ 2 cm	1		
> 2 cm	1.689	1.068–2.671	0.025
Skip metastasis			
No	1		
Yes	0.696	0.392–1.235	0.215
LN ratio (central)			
≤ 0.44	1		
> 0.44	2.492	1.508–4.118	< 0.001
LN ratio (lateral)			
≤ 0.29	1		
> 0.29	2.822	1.799–4.426	< 0.001
MRND type			
Ipsilateral	1		
Bilateral	2.158	1.267–3.676	0.004
Multifocality			
No	1		
Yes	1.735	1.131–2.662	0.012
Bilaterality			
No	1		
Yes	1.514	0.981–2.337	0.061
CLT			
NO	1		
Yes	0.542	0.305–0.962	0.037
ETE			
No/minor	1		
Gross	1.583	1.022–2.453	0.040
LVI			
No	1		
Yes	1.097	0.444–2.708	0.841
Stage			
I	1		
II	0.952	0.548–1.654	0.862
III	1.740	0.863–3.509	0.122

*CI* confidence interval, *LN* lymph node, *MRND* modified radical neck dissection, *CLT* chronic lymphocytic thyroiditis, *ETE* extrathyroidal extension, *LVI* lymphovascular invasion

In multivariate analysis, LN ratio > 0.44 in the central compartment (vs. ≤ 0.44; hazard ratio [HR], 1.890; 95% confidence interval [CI], 1.124–3.178; *p* = 0.015), LN ratio > 0.29 in the lateral compartment (vs. ≤ 0.29; HR, 2.351; 95% CI, 1.477–3.743; *p* < 0.001), and multifocality (vs. no multifocality; HR, 1.583; 95% CI, 1.030–2.431; *p* = 0.036) were associated with worse RFS (Table 5).

**Table 5:** Multivariate analysis of risk factors for recurrence

	Exp (B)	95% CI for Exp (B)	*p*
Main tumor size			
≤ 2 cm	1		
> 2 cm	1.480	0.931–2.355	0.098
LN ratio (central)			
≤ 0.44	1		
> 0.44	1.890	1.124–3.178	0.016
LN ratio(lateral)			
≤ 0.29	1		
> 0.29	2.351	1.477–3.743	< 0.001
Multifocality			
No	1		
Yes	1.583	1.030–2.431	0.036
CLT			
No	1		
yes	0.644	0.362–1.149	0.136
MRND type			
Ipsilateral	1		
Bilateral	1.335	0.744–2.394	0.332
ETE			
No	1		
Gross	1.308	0.824–2.076	0.255

*CI* confidence interval, *LN* lymph node, *CLT* chronic lymphocytic thyroiditis, *MRND* modified radical neck dissection, *ETE* extrathyroidal extension

### Postoperative complications

Of the 390 patients, the incidence of transient and permanent hypoparathyroidism were 16 (4.1%) and 3 (0.8%) patients, respectively. We observed transient and permanent recurrent laryngeal nerve palsy in 23 (5.9%) and 14 (3.6%) patients (Table 6). Among 29 patients with invasion to recurrent laryngeal nerve, 24 patients underwent shaving operation and 5 patients underwent re-anastomosis of recurrent laryngeal nerve; no patients underwent concurrent tracheostomy. Two patients underwent reoperation due to postoperative bleeding during admission after initial surgery; two patients showed chyle leakage, one of these patients underwent operative treatment and the remaining patient recovered after conservative management.

**Table 6:** Postoperative complications

	Number (%)
Hypoparathyroidism	
Transient	16 (4.1)
Permanent	3 (0.8)
Recurrent laryngeal nerve injury	
Transient	23 (5.9)
Permanent	14 (3.6)
Postoperative bleeding	2 (0.5)
Chyle leakage	2 (0.5)

## Discussion

Among PTC patients with N1b in this study, 44 (12.7%) had contralateral neck metastatic LNs. Contralateral neck LN metastasis was associated with male gender, more than 2 cm size of main tumor, a high number of metastatic central LN, multifocality and bilaterality of the tumors, and gross ETE. However, there was no significant relationship between type of MRND (ipsilateral MRND vs. bilateral MRND) and recurrence. LN ratio in central and lateral compartment, and multifocality of tumors were independent prognostic factors in N1b PTC patients.

According to ATA guidelines, prophylactic central LN dissection should be considered in patients with clinically central node-negative who have advanced primary tumors (T3 or T4) or clinically N1b [8]. However, prophylactic MRND is not recommended if the patients had no evidence of FANC or Tg washout measurement in the lateral compartment [8]. The sensitivity of US detection of lateral neck LN is higher than that of central LN [11]. Meticulous evaluation of lateral neck LN using US is needed for PTC patients during the preoperative evaluation period. In a study of 135 PTC patients who underwent bilateral neck dissection, the authors found that bilaterality of tumors and tumors arising in the isthmus were associated with bilateral LN metastasis [12]. They also demonstrated that contralateral neck LN metastasis was significantly correlated with clinically node-positive in the ipsilateral neck and contralateral paratracheal LN metastasis. In another study of 1776 PTC patients who underwent thyroidectomy and ipsilateral MRND during mean follow-up of 12.1 years, 32 (1.8%) patients recurred with contralateral neck LN [13]. They concluded that risk factors for contralateral neck LN were male gender, more than 2 cm size of primary tumor, ETE, and the presence of gross nodal metastasis at the initial surgery. Therefore, they suggested that patients with the abovementioned factors may be recommended for bilateral MRND. Although N1b PTC patients with a high number of metastatic central LN, multifocality, or bilaterality, as well as those with bigger tumor or gross ETE, tended to have contralateral neck LN metastasis in this study, further study is needed to demonstrate the execution of both MRND.

Several studies revealed that the number of metastatic LN at diagnosis were associated with recurrence in PTC [1, 14]. In addition, some studies found that LN ratio (the number of metastatic LN divided by the number of harvested LN) is related to post-treatment recurrence. A study of 198 PTC patients who underwent total thyroidectomy and neck dissection concluded that patients with LN ratio ≥ 0.3 had 3.4 times higher risk of persistent or recurrent disease than did those with ratio of 0 [15]. Schneider et al. reported that patients with LN ratio more than 0.7 showed significantly worse disease-free survival rates compared with those with ratio below 0.7 [16]. However, Lee et al. reported that central plus lateral LN ratio did not have an association with recurrence in patients who underwent therapeutic central and lateral neck dissection [17]. The present study separated LN status based on the location of metastatic LN: central compartment or lateral compartment. Skip metastasis, N1b without central LN metastasis, was not associated with recurrence. LN ratio > 0.44 in the central compartment and LN ratio > 0.29 in the lateral compartment were independent prognostic factors for poor RFS in patients with N1b disease. Even though the LN ratio in PTC is a useful prognostic factor for disease-free survival, multicenter studies are required to set optimal cutoffs, standardize the number and surgical extent, and supplement TNM staging.

Shattuck et al. described that individual tumor foci in multifocal PTC originate from discrete tumors independently [18]. Another study demonstrated that multifocal PTC stems from the same clone; therefore, it is important for intrathyroidal metastasis in PTC [19]. Although the reason of multifocality in PTC is not clear, multifocal PTC is not a rare event. Indeed, there is still controversy regarding multifocality in PTC and survival outcomes. Some study has reported that multifocality is not associated with recurrence [20]. Another study showed that bilaterality rather than unilateral multifocality of PTC was proven to be an independent risk factor for locoregional recurrence, distant metastasis, and cancer death [21]. On the other hand, Lin et al. revealed that multifocal PTC patients have higher recurrence rate and advanced TNM stage compared to solitary PTC patients [22]. Also, some authors described that patients with multifocal micro PTC were observed to have 5.6-fold higher LN recurrence [23]. Another study showed that the number of multifocal tumors rather than the location is significant predictive factor for disease recurrence [24]. This study showed 36.4% multifocality and 32.1% bilaterality in patients with N1b. The patients who underwent bilateral MRND tended to have multifocality and bilaterality; however, only multifocality was related to RFS.

ETE is an important prognostic factor for survival outcomes in PTC. In the sixth AJCC, ETE was classified as minor and gross [25]; however, according to recent AJCC recommendations, minor ETE was removed from the definition of T3 disease [3]. Therefore, tumors > 4 cm in greatest dimension limited to the thyroid gland is considered T3a and gross ETE with invasion only to the strap muscles is considered T3b [3]. This study did not include patients with T4b because complete resection was not achieved. The patients with gross ETE were associated with contralateral neck LN metastasis and poor RFS in univariate analysis; however, there was no statistically significance between gross ETE and RFS in multivariate analysis.

CLT is an autoimmune disease characterized by fibrosis, atrophy, and lymphocyte infiltration in thyroid tissue. CLT exhibits a thyroid-specific antigen that is represented in thyroid tumor and may be involved in the destruction of thyroid cancer. Several investigations reported that PTC patients with coexisting CLT have lower recurrence rate and better overall survival due to control of tumor growth and proliferation [26, 27]. This study showed that patients with CLT have lower recurrence rates in univariate analysis; however, there is no relation between CLT and recurrence in multivariate analysis.

In terms of the location of metastatic LN, Ito et al. revealed that the 621 N1b PTC patients with less than 3 cm metastatic lateral LN, less than five metastatic lateral LNs, or without extranodal extension had similar survival outcomes compared with those N1a PTC patients [10]. Several studies found that the location of metastatic LN rather than the number is useful for predicting risk of recurrence or distant metastasis; therefore, the distinction between N1a and N1b is extremely important for postoperative management in PTC patients [28, 29]. Even though the location of metastatic LN is not reflected in TNM stage, it is a powerful factor that affects disease-specific survival to decide the surgical extent.

According to the ATA guidelines, neck US should be performed at 6–12 months after initial surgery and then periodically depending on the risk of recurrence and Tg status [8]. A considerable number of patients had a recurrence within 5 years in this study; thus, we suggest that patients who had undergone MRND should be checked via neck US every 6 months at least for 5 years. In addition, neck CT is useful for detection of especially suspicious central LNs. Therefore, we suggest that neck CT should be performed periodically for patients with negative neck US and high level of Tg.

This study has several limitations. Study design was retrospective and conducted at a single institution. In order to determine laterality of lateral LN metastasis if the patients who underwent ipsilateral MRND had bilaterality of tumors, it was decided by the location of the bigger tumor. Therefore, selection bias is reflected in decisions regarding laterality. In addition, the decision of surgical extent might be intervened by interpretation of radiologists and surgeons of preoperative imaging modalities like neck US and neck CT. We did not consider patients with biochemical incomplete response that related to Tg and anti-Tg measurement. We are collecting more sufficient clinicopathological data for long-term follow-up of patients who underwent total thyroidectomy and MRND.

## Conclusions

The patients who underwent ipsilateral MRND or bilateral MRND have the same N stage. The surgical extent of lateral neck compartment is not reflected in TNM stage. However, meticulous preoperative evaluation of contralateral LN is needed to avoid residual or persistent disease during postoperative follow-up. The factors that associated with contralateral LN metastasis were male gender, more than 2 cm size of main tumor, multifocality, bilaterality, and ETE. Recurrence in N1b PTC patients is predicted by central neck LN ratio > 0.44, lateral neck LN ratio > 0.29, and multifocality of tumors. We suggest that patients with these factors should receive short-term follow-up using image modalities like US and CT.

## Abbreviations

AJCC: American Joint Committee on Cancer
ATA: American Thyroid Association
CI: Confidence interval
CLN: Central neck lymph node
CLT: Chronic lymphocytic thyroiditis
CT: Computed tomography
ETE: Extrathyroidal extension
FANC: Fine needle aspiration cytology
HR: Hazard ratio
LLN: Lateral neck lymph node
LN: Lymph node
LVI: Lymphovascular invasion
MRND: Modified radical neck dissection
PTC: Papillary thyroid cancer
RFS: Recurrence-free survival
SD: Standard deviation
US: Ultrasonography
